# Validation of brief screening tools for depressive and alcohol use disorders among TB and HIV patients in primary care in Zambia

**DOI:** 10.1186/1471-244X-11-75

**Published:** 2011-05-04

**Authors:** Nathaniel Chishinga, Eugene Kinyanda, Helen A Weiss, Vikram Patel, Helen Ayles, Soraya Seedat

**Affiliations:** 1 Zambia AIDS-Related TB Project, School of Medicine, Ridgeway campus, Lusaka, Zambia; 2 Department of Clinical Research, London School of Hygiene & Tropical Medicine, London, UK; 3 Medical Research Council/Uganda Virus Research Institute, Unit on AIDS, Entebbe, Uganda; 4 Medical Research Council Tropical Epidemiology Group, Department of Infectious Disease Epidemiology, London School of Hygiene & Tropical Medicine, London, UK; 5 Centre for Global Mental Health, London School of Hygiene & Tropical Medicine, UK; 6 Medical Research Council Anxiety and Stress Disorders Unit, Department of Psychiatry, University of Stellenbosch, Cape Town, South Africa

## Abstract

**Background:**

This study was conducted to evaluate the diagnostic accuracy and determine the optimum cut-off scores for clinical use of the Center for Epidemiological Studies Depression scale (CES-D) and Alcohol Use Disorders Identification Test (AUDIT) against a reference psychiatric diagnostic interview, in TB and anti-retroviral therapy (ART) patients in primary care in Zambia.

**Methods:**

This was a cross-sectional study in 16 primary level care clinics. Consecutive sampling was used to select 649 participants who started TB treatment or ART in the preceding month. Participants were first interviewed using the CES-D and AUDIT, and subsequently with a psychiatric diagnostic interview for current major depressive disorder (MDD) and alcohol use disorders (AUDs) using the Mini-International Neuropsychiatric Interview (MINI). The diagnostic accuracy was calculated using the Area Under the Receiver Operating Characteristic curve (AUROC). The optimum cut-off scores for clinical use were calculated using sensitivity and positive predictive value (PPV).

**Results:**

The CES-D and AUDIT had high internal consistency (Cronbach's alpha = 0.84; 0.98 respectively). Confirmatory factor analysis showed that the four-factor CES-D model was not a good fit for the data (Tucker-Lewis Fit Index (TLI) = 0.86; standardized root-mean square residual (SRMR) = 0.06) while the two-factor AUDIT model fitted the data well (TFI = 0.99; SRMR = 0.04). Both the CES-D and AUDIT demonstrated good discriminatory ability in detecting MINI-defined current MDDs and AUDs (AUROC for CES-D = 0.78; AUDIT = 0.98 for women and 0.75 for men). The optimum CES-D cut-off score in screening for current MDD was 22 (sensitivity 73%, PPV 76%) while that of the AUDIT in screening for AUD was 24 for women (sensitivity 60%, PPV 60%), and 20 for men (sensitivity 55%, PPV 50%).

**Conclusions:**

The CES-D and AUDIT showed high discriminatory ability in measuring MINI-defined current MDD and AUD respectively. They are suitable mental health screening tools for use among TB and ART patients in primary care in Zambia.

## Background

Mental health disorders, human immunodeficiency virus (HIV) and tuberculosis (TB) have a profound impact on public health in sub-Saharan Africa [1], yet there are limited data on the interaction between major depressive disorders (MDDs), alcohol use disorders (AUDs) with HIV [2] and TB in this region. Many sub-Saharan African countries carry a high burden of HIV [3] and alcohol-related morbidity and mortality [4,5]. For example, the prevalence of MDDs among HIV positive individuals has been estimated as 43.7% in South Africa [6], 71.3% in Zimbabwe [7] and 47% in Uganda [8].

The causal relationships between mental disorders and HIV are complex [1]. MDD [9] and hazardous alcohol consumption [10,11] are associated with high risk of HIV acquisition and transmission, and with poorer adherence to anti-retroviral therapy (ART) [12] and TB treatment [13]. Conversely diagnosis with HIV increases risk of depression and alcohol abuse [14]. Neuropsychiatric complications of HIV include HIV encephalopathy, depression, mania, cognitive disorders, and frank dementia, alone or in combination. AUDs [15] and MDDs have also been found to be associated with HIV disease progression [16,17].

The TB and HIV burden is high in primary health care (PHC) facilities in Zambia [18]. The HIV prevalence in Zambian adults is estimated to be 14.3% [19] and the estimated prevalence of tuberculosis in Zambia is 387 per 100 000 population [20] with approximately 70% of TB infection in Zambia related to HIV [21]. A study conducted in Zambia in four PHC facilities found the prevalence of common mental disorders to be 13.6% (diagnosed by DSM-IV criteria) [22]. A population-based HIV survey in Zambia found the prevalence of mental distress in HIV-infected individuals to be 20.8% [23].

The diagnosis of common mental disorders in TB and HIV-infected patients in PHC facilities is essential for improving population health [24]. However, like many low income countries, Zambia has a high attrition of health workers [25], and few are skilled in detecting MDDs and AUDs. In the effort to mitigate the health worker crisis in Africa, TB and HIV programs are task shifting care to lower cadre staff [26]. TB and HIV programs also need to integrate mental health [24] and include assessment of mental health disorders and their appropriate management [14]. Introducing screening tools for mental health that can be used by non-specialists or lay workers could make a dramatic contribution to the health sector's ability to identify those in need of mental health support. Such screening tools however need to be validated for the populations in which they are to be used.

The aim of this study is to evaluate the diagnostic accuracy and determine the optimum cut-off scores for clinical use of the Center for Epidemiological Studies Depression scale (CES-D) [27] and the Alcohol Use Disorders Identification Test (AUDIT) [28] in detecting DSM-IV current MDD and AUD respectively, among TB patients on TB treatment and HIV patients on ART in PHC settings in Zambia. DSM-IV criteria for MDDs and AUDs were assessed using the Mini-International Neuropsychiatric Interview (MINI) [29] as the reference.

## Methods

### Study design and setting

This was a cross-sectional study under the auspices of the Zambia AIDS-Related TB (ZAMBART) Project. The study was conducted in PHC centres that provide both TB and HIV diagnoses and treatment. The TB/ART clinics within the PHC centres are serviced by clinical officers and nurses, many of whom have limited training in screening for MDDs and AUDs. These centres cover both urban and rural settings and are the first point of entry in the referral process for the majority of TB and HIV patients in the early stages of disease. Sixteen PHC centres were selected for the study on the basis of high TB and HIV prevalence [30] and these are distributed in seven districts, of which Lusaka (the capital city) contains four centres and the other six districts have two centres each. The size of the catchment area for these PHC centres varied from 25,000 inhabitants in rural communities to 147,000 inhabitants in urban communities.

### Training of the Field staff

Sixteen lay research assistants and ten mental health clinical assistants (mental health workers with a diploma in mental health), recruited from the study communities, were trained separately over two days. On the first day, the lay research assistants were trained on how to screen for common mental disorders using the CES-D and AUDIT tools. On the second day the mental health clinical assistants were trained on how to diagnose common mental disorders using the MINI. The training for both groups also covered information on the study protocol, informed consent procedures, ethical considerations and data quality issues. The training was led by two psychiatrists, two TB/HIV specialists and a data manager. Inter-rater reliability assessments formed part of this training. The intra-class correlation value for interrater reliability of the lay research assistants was 0.98 while that of the mental health clinical assistants was 0.99. These results showed a high degree of inter-rater reliability in each group of field staff.

### Participants

Eligible participants were aged 16 years or older, attending the TB or ART clinics of one of the 16 PHCs, and had started TB treatment or ART during the month before the study. Patients with whom it was not possible to complete the informed consent procedures owing to serious medical illness were not eligible. Patients with dual TB and HIV infection were included, and data pertaining to both TB therapy and ART were documented.

### Procedures

A consecutive series of eligible patients were recruited from the PHC centres from December 2009 to January 2010. Participants were informed about the study and asked to provide informed consent after which data collection commenced in two separate interviews which were carried out on the same day of their routine clinic visits. The first interview was a screening interview conducted by the trained lay research assistants. Participants were screened for depression with the CES-D and excessive alcohol use with the AUDIT. Socio-demographic data and HIV/TB clinical data were also obtained in this interview. DSM IV criteria were assessed using the MINI in a second interview conducted by the trained mental health clinical assistants. Confirmatory clinical data on TB treatment and ART status were also obtained in this second interview from the participants' medical records. The trained mental health clinical assistants conducting the second interview with the MINI were blinded to all data collected by the trained lay research assistants conducting the first interview with the CES-D and AUDIT. We chose the CES-D and AUDIT because they are brief, easy to administer and have been widely used in cross-cultural studies, including African settings [31].

### Reference standard

The MINI was used as the 'gold-standard' in generating psychiatric diagnoses. The MINI is a short, structured diagnostic interview that was developed in 1990 by psychiatrists and clinicians in the United States and Europe for DSM-IV psychiatric disorders [29]. The MINI is divided into modules, each corresponding to a diagnostic category. For the purposes of this study, only modules covering current MDD and AUDs were selected. DSM-IV criteria have been used in previous studies in Zambia [22,23].

### Screening tools

The CES-D is a self-report scale with 20 items designed to measure depressive symptoms in general population samples. Each item is assigned a value 0-4. There are four items that are positive-worded that have to be reverse scored, before computing the total score by adding each of the 20 items. The minimum score is 0 and the maximum score is 60. The CES-D measures common symptoms of major depression, including depressive mood, feelings of guilt and worthlessness, psychomotor retardation, loss of appetite, and sleep disturbance within the week prior to the interview. A score of 16 and above in the general population suggests symptoms of depression [27]. Reliability, validity, and factor structure have been found to be similar across a wide variety of demographic characteristics in general population samples that have been tested [32,33]. In Uganda, the CES-D has been used to assess the prevalence of depression in HIV infected individuals, although it was not validated in this population [8].

The AUDIT was developed by the World Health Organisation (WHO) as a simple method of screening for excessive alcohol consumption in the past 12 months [28,34]. It consists of 10 questions on recent alcohol use (items 1-3), alcohol dependency syndromes (items 4-6) and alcohol-related problems (items 7-10). Each of the 10 questions is rated on a four-point scale. The total score ranges from 0 to 40. A total score of 8 or more is recommended as an indicator of hazardous drinking behaviour [34].The AUDIT was developed and validated in multinational samples involving Kenya [28] and has been validated in South Africa [31].

### Translation of the MINI and screening tools

The MINI, CES-D and AUDIT were translated to the dominant languages (Bemba, Nyanja, Tonga and Lozi) in the study communities. The translated questionnaires were forward-translated by professional translators working for the Zambia National Broadcasting Corporation (ZNBC). The forward-translated instruments were then back-translated into English by community representatives with experience in the translation of research questionnaires. Discrepancies in conceptual and semantic equivalence were resolved through an informal committee consensus approach with both forward and back-translators. Following on all this, all translated versions of the questionnaires were discussed by the research team; comprising members who were fluent in the native languages, until final versions of the questionnaires were agreed upon. These instruments were tested before use in the field.

### Definition of cases

The sample was categorised into cases and non-cases of psychiatric disorders based on the MINI outputs of (i) current MDD and (ii) AUD.

### Analysis

Data were analysed using Stata 11 (College Station, Texas, USA). Median total scores on the CES-D and AUDIT respectively were compared against MINI-defined diagnoses using the Wilcoxon rank-sum test. The internal consistency of these screening tools was assessed using Cronbach's alpha. We used confirmatory factor analysis to examine how a four-factor model of 'depressive symptoms', 'somatic symptoms', 'positive experiences' and 'interpersonal difficulties' for the CES-D [27], and a two-factor model of 'alcohol consumption' and 'alcohol related problems' for the AUDIT [35] fit the observed data. We used a combination of the chi-square to degrees of freedom ratio (χ^2^/df) of <2; Tucker-Lewis index (TLI) and Comparative fit index (CFI) of >0.95, and Standardized Root Mean-Square Residual (SRMR) of <0.08 as our rule of thumb for goodness of fit of the models [36].

Non-parametric area under the receiver operating characteristic curve (AUROC) analyses were performed to estimate the diagnostic accuracy of the screening tools [37]. Cut-off scores that simultaneously gave high sensitivity and high PPV were selected [38]. The data for the AUDIT was stratified by gender as previous research had shown that cut-off for the AUDIT was gender specific [39,40].

### Ethical Considerations

The study was approved by the University of Zambia Biomedical Research Ethics committee and endorsed by the Ministry of Health in Zambia. Written informed consent for participation and publication was obtained from the patients prior to the commencement of any study related procedures. Data were collected anonymously and all participants were identified by a unique study code. The questionnaires and electronic database were linked by these unique barcodes that were kept separately in a password protected database.

## Results

### Characteristics of the participants

**Figure 1:**
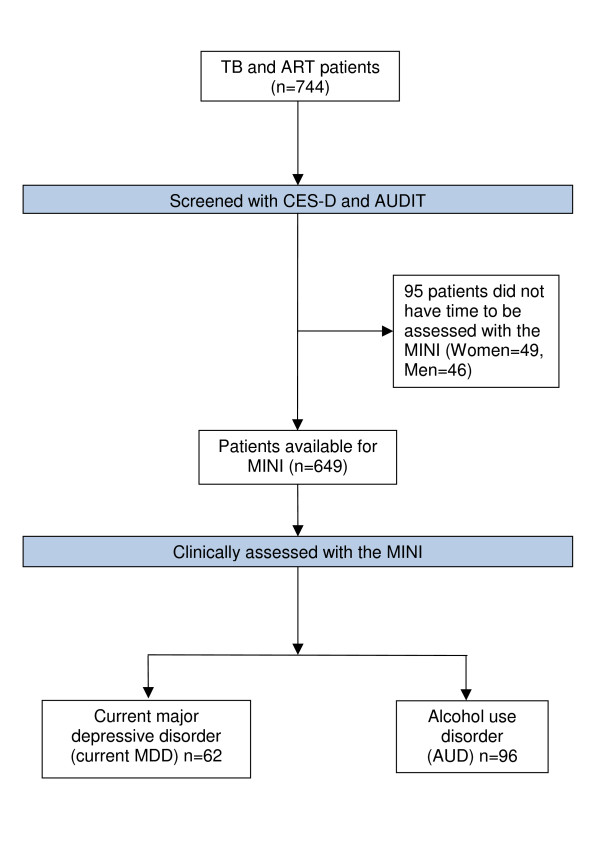
**Diagram of participant flow**.

Seven hundred and forty four patients participated in the first interview with the CES-D and AUDIT. Of these, 649 patients (87.2%) completed the MINI diagnostic interview (Figure 1). There was little evidence of a difference in age (p = 0.45), gender (p = 0.12), median CES-D score (p = 0.47) or median AUDIT score (p = 0.49) between those who did, and did not, complete the MINI diagnostic interview. Of the 649 participants who completed the MINI diagnostic interview, the majority (77%) were recruited at TB clinics, and of these, 54% were also HIV positive (Table 1).

**Table 1:** Characteristics of the study participants enrolled from 16 PHC centres in Zambia.

	Total (N = 649)
**Socio-demographics**	
Median age (years) (IQR)	33 (28-40)
Female (%)	286 (44.1)
Married (%)	
single	173 (26.6)
married	304 (46.8)
widowed	80 (12.3)
divorced	92 (14.2)
Education (%)	
No education	55 (8.5)
Primary	253 (39.0)
Secondary	341 (52.5)
Employed (%)	271 (41.8)
**Screening tools**	
Median CES-D score (IQR)	19 (15-26)
Median AUDIT score (IQR)	15 (10-23)
Median AUDIT score for Men (IQR)	16 (11-23)
Median AUDIT score for Women (IQR)	11 (5-22)

The median AUDIT score was higher for men than for women (median 16 vs. 11; p = 0.04).

MINI, Mini - International Neuropsychiatric Interview; IQR, inter-quartile range.

### Internal consistency

The internal consistency of the CES-D and AUDIT was high (Cronbach's α = 0.84 and 0.98 respectively). The goodness of fit indices for the CES-D suggest that the four-factor model did not fit the observed data well. Even though the χ^2^/df ratio was below 2 and SRMR was close to the desirable region (<0.08), the TLI and CFI for the four-factor CES-D model were below 0.95. The factor loadings for each CES-D item and the inter-correlation among the four factors were low (Table 2). The goodness of fit indices for the two-factor AUDIT shows that the χ^2^/df ratio, SRMR, TLI and CFI criterion were met. Thus the two-factor AUDIT model fits the observed data well. The factor loadings for each AUDIT item were high indicating that the correlation between each item and the respective latent factor was high (Table 3).

**Table 2:** Factor loadings matrices of a CES-D model, inter-correlation among factors and goodness of fit indices.

		Factor 1 depressed	Factor 2 Somatic	Factor 3 positive experiences	Factor 4 Interpersonal difficulties
**Item**					
1.	I felt that I could not shake off the blues even with help from my family or friends.	0.48			
2.	I felt depressed	0.58			
3.	I thought my life had been a failure	0.46			
4.	I felt fearful.	0.60			
5.	I felt lonely.	0.55			
6.	I had crying spells.	0.59			
7.	I felt sad.	0.66			
8.	I was bothered by things that usually don't bother me.		0.33		
9.	I did not feel like eating; my appetite was poor.		0.41		
10.	I had trouble keeping my mind on what I was doing.		0.45		
11.	I felt that everything I did was an effort.		0.33		
12.	My sleep was restless.		0.49		
13.	I talked less than usual.		0.45		
14.	I could not get going.		0.55		
15.	I felt that I was just as good as other people.			0.38	
16.	I felt hopeful about the future.			0.35	
17.	I was happy.			0.56	
18.	I enjoyed life.			0.56	
19.	People were unfriendly				0.69
20.	I felt that people disliked me.				0.69
**Inter-factor correlation**					
	Factor 1	1.00			
	Factor 2	0.21	1.00		
	Factor 3	0.15	0.18	1.00	
	Factor 4	0.20	0.16	0.12	1.00
**Goodness of Fit Indices**					
	Chi-square/degrees of freedom (χ^2^/df ratio)	1.85			
	Standardized Root Mean-Square Residual (SRMR)	0.06			
	Tucker-Lewis Index (TLI)	0.88			
	Comparative Fit Index (CFI)	0.86			

**Table 3:** Factor loadings matrices of an AUDIT model, inter-correlation among factors and goodness of fit indices.

		Factor 1 Alcohol consumption	Factor 2 Alcohol related problems
**Item**			
1.	How often do you have a drink containing alcohol?	0.98	
2.	How many drinks containing alcohol do you have on a typical day when you are drinking?	0.97	
3.	How often do you have six or more drinks on one occasion?	0.98	
4.	How often during the last year have you found that you were not able to stop drinking when you started?		0.98
5.	How often during the last year have you failed to do what was normally expected of you because of drinking?		0.99
6.	How often during the last year have you needed a first drink in the morning to get yourself going after a heavy drinking session?		0.98
7.	How often during the last year have you had a feeling of guilt or remorse after drinking?		0.96
8.	How often during the last year have you been unable to remember what happened the night before because of your drinking?		0.98
9.	Have you or someone else been injured because of your drinking?		0.98
10.	Has a relative, friend, doctor or health worker been concerned about your drinking or suggested you cut down?		0.96
**Inter-Factor Correlations **			
	Factor 1	1.00	
	Factor 2	0.55	1.00
**Goodness of Fit Indices**			
	Chi-square/degrees of freedom (χ^2^/df ratio)	1.14	
	Standardized Root Mean Square Residual (SRMR)	0.04	
	Tucker-Lewis Index (TLI)	0.99	
	Comparative Fit Index (CFI)	0.99	

### Case detection properties

CES-D scores tended to be higher among MINI-defined current MDD cases than non-MDD cases (median 28 vs. 18; p < 0.001). Similarly, the AUDIT scores were higher among MINI-defined AUD cases than non-cases (median 22 vs.12; p < 0.001). The AUDIT scores were also higher among MINI-defined AUD cases than non-cases for women (median 24 vs.10; P < 0.001) and men (median 20 vs.13; p < 0.001) respectively.

**Figure 2:**
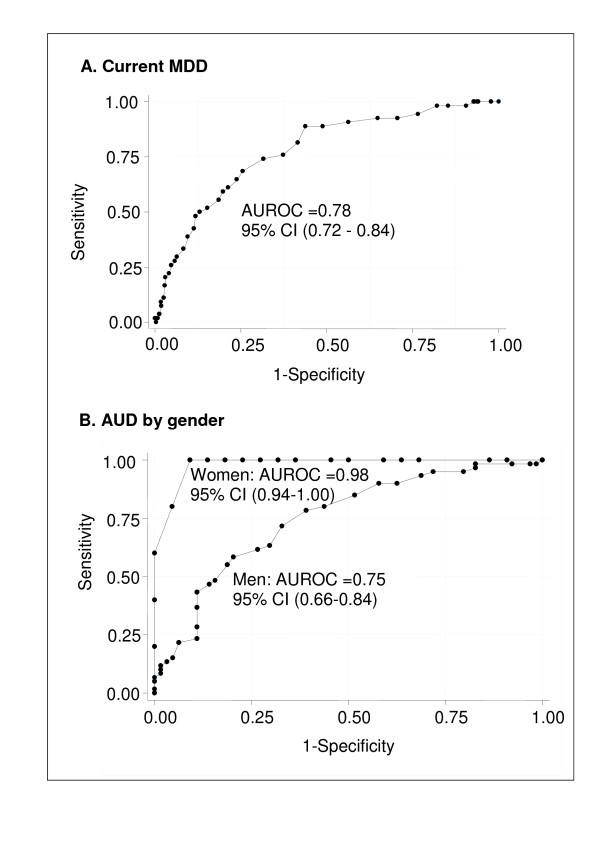
**Area under the receiver operating characteristic curve (AUROC) with 95% confidence intervals (95% CI) for the CES-D and AUDIT total scores for diagnosis of current MDD (A) and AUD (B)**. The AUROC for AUD was significantly different between women and men (P < 0.0001).

The CES-D and AUDIT showed good discrimination in detecting current MDD and AUD cases from non-cases respectively (AUROC for CES-D = 0.78, and for AUDIT = 0.98 for women and 0.75 for men respectively), indicating that these were accurate screening tools. The difference in performance of the AUDIT was significantly better for women than for men (p < 0.0001) (Figure 2).

**Figure 3:**
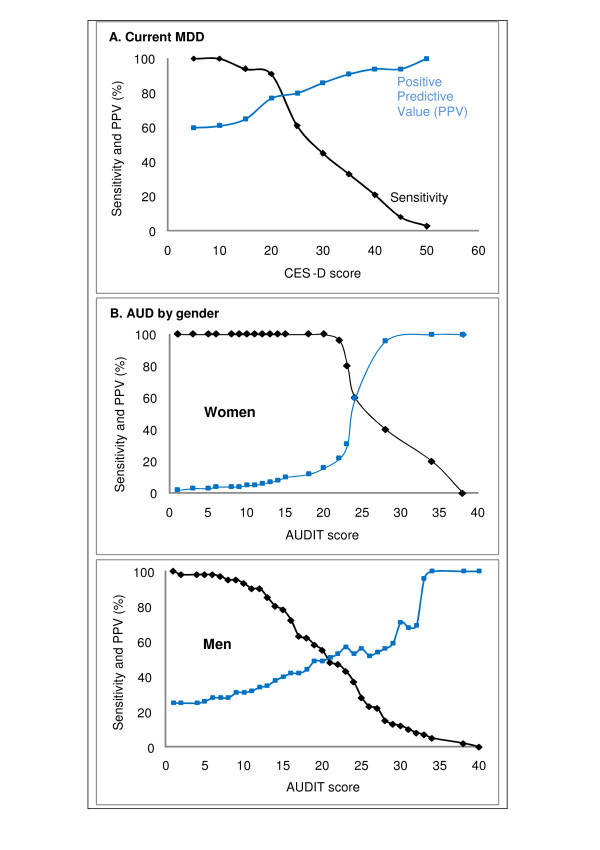
**Sensitivities and positive predictive values for the CES-D and AUDIT by cut-off scores, for diagnosis of current MDD (A) and AUD (B)**.

For each CES-D and AUDIT, cut-off points, sensitivity and PPV were obtained (Figure 3). The optimum cut-off score of the CES-D in screening for current MDD was 22. This achieved a sensitivity of 73% and PPV of 76% (Figure 3A). For the AUDIT, the optimum cut-off score for screening AUDs was 24 for women (sensitivity of 60% and PPV of 60%), and 20 for men (sensitivity of 55% and PPV of 50%) (Figure 3B).

## Discussion

This study shows that the CES-D and AUDIT are reliable and valid instruments to use among TB and HIV patients in primary care. Using a singular construct to test for internal consistency, we found that the Cronbach's alpha was 0.84 for the CES-D and 0.98 for the AUDIT. This indicates that the participants showed adequate consistency in their responses. These high estimates are similar to previous studies performed on the CES-D [27] and the AUDIT [41]. The four-factor model for the CES-D did not fit the data well. This means that the latent four factors in the CES-D were mis-specified. The two-factor model for the AUDIT showed the desired goodness of fit. This indicates that the two factors in the AUDIT could be considered as subscales.

The AUROCs for the CES-D and AUDIT (for both women and men) were high in detecting current MDD and AUDs from non-cases respectively. These findings are in keeping with a validation study conducted among HIV-infected person in South Africa that found the CES-D and AUDIT performed well in accurately discriminating MINI-defined current MDD (AUROC curve 0.76) and AUD (AUROC 0.96) respectively [31]. The better accuracy of the AUDIT in women agrees with other studies [39,40].

A highly sensitive test is needed for screening examinations in routine clinical care to identify potential cases, while a highly specific test is best in a confirmatory role. Of the cases identified by a screening test, few should be false positives (i.e. have high PPV) so that the expense and morbidity of further evaluation of false positive results are reduced in settings that already have limited resources [38]. In our study, both the CES-D and AUDIT met the criterion of having cut-off scores that simultaneously have moderate to high sensitivities and PPVs. At a cut-off of 22, the CES-D yielded a sensitivity of 73% and PPV of 76% for current MDD. Similarly, at a cut-of score of 24 for women and 20 for men, the AUDIT yielded a sensitivity of 60% and PPV of 60% for women, and a sensitivity of 55% and PPV of 50% men for AUDs. The sensitivities of the AUDIT were moderate (55% sensitivity for men; 60% for women), meaning that 55-60% of the true AUD cases were identified. Also, the PPVs of the AUDIT were moderate (50% for men; 60% for women), indicating that those who screened positive about half were actually cases. The cut-offs were high compared to the CES-D cut-off of 16 [27] and AUDIT cut-offs of 8 [34] found in the general population. This discrepancy may indicate that our study population may have a greater likelihood of having current MDD and hazardous alcohol drinking than the general population. The high cut-offs may also reflect greater severity of current MDD and alcohol problems among our study participants; these may need intensive interventions.

Despite the available infrastructure for psychiatric admissions and outpatient care, most health facilities in Zambia do not have adequate health workers to treat depression and alcohol use disorders. We therefore recommend that individuals with high AUDIT or CES-D scores in this setting be offered treatment in accordance with the WHO Mental Health Gap Action Programme (mhGAP) [42]. The mhGAP is a tool designed by the WHO to be used in PHC settings where health workers have limited training in Psychiatry. The mhGAP guidelines for depression include offering psychoeducation to the patient on the importance of continuing activities that used to be interesting for them and maintaining regular sleep cycles; physical activity; social activity and scheduled visits with the primary care professional when thoughts of suicide or self-harm arise. The guidelines also indicate the need to address the current psycho-social stressors for the patient by giving them the opportunity to talk about what they think are the causes of the symptoms they have, and by identifying family members who could help them solve these stressors. Furthermore, they indicate the need to identify the patient's prior physical activities, so that if these activities are re-initiated, they would have the potential for providing psycho-social support. Lastly, the guidelines indicate that if cognitive behaviour therapy (CBT) is available, it should be used on patient during scheduled visits at the clinic.

The mhGAP guidelines for those with alcohol use disorders include discussing with the patient the short and long-term risks of continued use of alcohol; asking about other substance use; having a discussion about their reasons for alcohol use, and providing examples of ways that the harmful or hazardous use of alcohol could be reduced. If the patient fails to respond or is suspected to have alcohol dependence, they should be referred to a specialist for further diagnostic evaluation and possible treatment for alcohol dependence. For those who score lower on the AUDIT, a Brief Drinker Profile [43] can be performed which measures quality and frequency of drinking in the previous month, and advice given on the effects of alcohol consumption on medication.

Generalisability of our findings is limited to TB and HIV patients on treatment in PHC centres. Further measures of depression and AUDs at a general population level in Zambia may be needed so that the diagnostic accuracy of CES-D and AUDIT test results among patients with depression and AUDs can be compared to those without these disorders.

## Conclusions

The CES-D and AUDIT showed high discriminatory ability in measuring MINI-defined current MDD and AUD respectively. The CES-D showed high sensitivity and PPV while the AUDIT showed moderate sensitivity and PPV in men and women, indicating that these are suitable tools for screening current MDD and AUD among TB and ART patients in PHC settings where resources are limited.

## Competing interests

The authors declare that they have no competing interests.

## Authors' contributions

NC, HA, EK, and SS were involved in the conception and design of the study. NC supervised the data collection. NC and HAW did the data analysis. NC wrote the first draft of the manuscript. VP gave direction to the manuscript. All authors contributed to the interpretation of data; revising the manuscript critically for important intellectual content; and final approval of the version to be published.

## Pre-publication history

The pre-publication history for this paper can be accessed here:

http://www.biomedcentral.com/1471-244X/11/75/prepub
